# Apolipoprotein E Polymorphism and Colorectal Neoplasm: Results from a Meta-Analysis

**DOI:** 10.1371/journal.pone.0102477

**Published:** 2014-07-16

**Authors:** Yun Tian, Jirong Wang, Ying Ye, Liqun Sun, Yingrui Fan, Li Wang, Juan Li, Zhaoxia Wang, Keming Wang

**Affiliations:** 1 Department of Oncology, the Second Affiliated Hospital of Nanjing Medical University, Nanjing, Jiangsu, PR China; 2 Emergency Center, Affiliated Hospital of Xuzhou Medical College, Xuzhou, Jiangsu, PR China; 3 Department of Intensive Care Unit, the Second Affiliated Hospital of Nanjing Medical University, Nanjing, Jiangsu, PR China; University of Aberdeen, United Kingdom

## Abstract

To investigate the relationship of Apolipoprotein E (*APOE*) gene polymorphism to colorectal neoplasia (CRN), we performed a systematic review and meta-analysis. Eligible studies were identified through a systematic literature review from PubMed, EMBASE, and the Science Citation Index up to February 2014. A combined analysis was performed, followed by a subgroup analyses stratified by the study design. We used data collected from 8 prospective studies involving respectively a total of 9243 participants and 4310 CRN cases which including 438 patients with colorectal adenoma (CRA), and 3873 patients with colorectal carcinoma (CRC). The pooled data from this meta-analysis indicated there was no significant association between *APOE* polymorphism and CRN (ε2: P = 0.51, OR 1.04 95% CI 0.93 to 1.16; ε4: P = 0.72, OR 0.98 95% CI 0.90 to 1.07). Interestingly, subgroup analysis demonstrated there was a significant decreased risk for proximal CRN in patients with *APOE* ε4 (P = 0.0007, OR 0.52 95% CI 0.35 to 0.76). Data showed no significant association between *APOE* genotype and overall CRN. However, compared with those carry APOE ε3 alleles, persons with *APOE* ε4 genotype have significant decreased risk suffering from proximal CRN but not from distal CRN.

## Introduction

Colorectal neoplasm (CRN) is an epithelial polyps which resulted from abnormal proliferation of colonic epithelial cells. Colorectal adenoma (CRA) is recognized as the well-established precursors of colorectal cancer (CRC) [1], [2], [3]. Generally, CRA can develop into CRC through an adenoma to carcinoma sequence [4]. CRC is the third most common cancer in world-wide, accounting for 8% of all cancers [5]. For the past decades, the mortality rate of CRC has been declined because of screening colonoscopy. Despite the success of screening colonoscopy for CRC prevention, people will be benefitted by identifying additional risk factors for CRC that might facilitate novel prevention strategies.

Apolipoprotein E (*APOE*) gene polymorphism is demonstrated to be a major factor in lipid metabolism. It is recognized recently that polymorphism of gene encoding *APOE* to be potential risk factor for CRN [6]. The human *APOE* gene, which produces three distinct protein isoforms: wild-type *APOE* E3 (112Cys/158Arg), *APOE* E2 (112Cys/158Cys), and *APOE* E4 (112Arg/158Arg), is found located on chromosome 19 [7], [8]. Of these three isoforms, the most seen one is *APOE* E3 with a frequency of approximately 70% to 80%, while each isoform of these isoforms has its unique receptor binding activity individually [9], [10]. *APOE* is demonstrated to have the ligand for receptors of the low-density lipoprotein (LDL) receptor family. In addition, *APOE* plays an important role in the synthesis of very low-density lipoprotein (VLDL) and the process of the VLDL remnants hydrolysis [11]. Accumulated data indicated persons with the E2 allele presented defective receptor-binding ability, had lower plasma cholesterol levels and higher triglyceride levels. However, people with the E4 allele were found have a higher serum level of cholesterol [12], [13]. Furthermore, *APOE* ε4 has been implicated in coronary heart disease (CHD), age-related cognitive decline, and Alzheimer’s disease [14], [15], [16]. It is also demonstrated by a meta-analysis that people with the ε4 allele *APOE* genotypes had a 42% increased risk of CHD than those with the ε3 allele [10]. However, the association between *APOE* genotype and other disease such as CRN is less clear.

Recent studies indicated that *APOE* may show its activity in CRC development by function in β-catenin localisation, tumor cell metastasis, DNA synthesis, antioxidant abilities, cell proliferation, and angiogenesis [17], [18], [19], [20]. *APOE* also plays a major role in altering metabolism of cholesterol and bile acids, modulating angiogenesis, carcinogenic cell growth as well as metastasis [20], [21], [22], [23]. Many studies have attempted to clarify the relationship of *APOE* polymorphism and colorectal tumor risk although the conclusions are still contradictory. A study reported that *APOE* E4 may be a protective factor for CRC while those with the E2 or E3 genotype had an increased risk of colon carcinoma in male [24]. In addition, a study from Brazil found the ε4 genotype only presents in controls [25]. A study from China found subjects with *APOE* ε3/ε4 genotype have lower risk suffering from CRA than those with other genotypes [26]. In addition, Mrkonjic et al reported no significant differences in *APOE* genotype frequencies were observed between CRC cases and unaffected controls [6]. Furthermore, another large sample case-control study did not detect any significant associations between *APOE* genotype and rectal cancer [27].

As the contrary conclusions of *APOE* gene polymorphism in CRN, for the first time, we performed a systematic review and meta-analysis focusing on the *APOE* genotype of possible relevance to colorectal carcinogenesis.

## Materials and Methods

### Search strategy

The published Quality of Reporting of Meta-analysis (QUOROM statement) was followed in our study [28]. Electronic databases which included PubMed, EMBASE, and the Science Citation Index were searched for identification of studies on *APOE* polymorphisms and CRN published up to February 2014. The following search terms were used: “colorectal neoplasm”, “colorectal cancer”, “colorectal adenoma”, “polymorphisms”, “apolipoprotein E” or “*Apo E*” or “*apoE*” or “*APOE*”. Additionally, the reference lists of relevant publications were also screened for additional relevant studies. As a prerequisite, only those studies published in English language and focused on human subjects were identified.

### Inclusion criteria

Studies included in this meta-analysis according to following criteria: 1) evaluation of *APOE* polymorphism in association with CRN (including CRC and CRA); 2) study design was “cohort” or “prospective” or “follow-up” or “cross sectional” or “case–cohort” or “nested case–control”; 3) allele counts of *APOE* polymorphisms of cases and controls could be extracted. Studies were excluded if the data were not sufficient to perform meta-analysis. In addition, review articles or published abstracts from meeting were excluded. Furthermore, articles selected for meta-analysis had no overlap of subjects with other studies.

### Data extraction

Studies included in this meta-analysis were reviewed twice by using a standardized form for data extraction. Two authors (Y. Tian and J. Wang) independently carefully drew all the studies. Data were collected on the first author’s name, year of publication, source of control group (population based or hospital based), study design, ethnicity of patients and controls, country of origin, and numbers of *APOE* alleles among patients and controls. The data was extracted from each publication by a standardized protocol.

### Statistical analysis

The associations between *APOE* polymorphisms and CRN were evaluated by using the software Review Manager (V5.1) for windows (Oxford, England, UK). We first analyzed the risk of the ε2 and ε4 alleles compared with the wild-type ε3 allele for the development of CRN. Second, the association between *APOE* polymorphisms and susceptibility to CRC was estimated. Finally, we performed the meta-analysis of the relationship of the ε2 carriers and ε4 carriers and CRA risk. A statistical test for heterogeneity was performed based on the Q statistic test with a p-value less than 0.05 was considered as significant heterogeneity between studies to account for the possibility of heterogeneity across studies [29]. The data were analyzed by using both fixed effects and random effects models. The fixed-effects method by Mantel and Haenszel was used in the condition of no significant heterogeneity [30], while the random-effects method by DerSimonian and Laird [31] was more appropriate when heterogeneity was present. Publication bias analysis was measured using Stata 11.0 (Stata Corp, College Station, TX) with Begg and Egger tests [32], [33].

## Results

### Study characteristics

**Figure 1:**
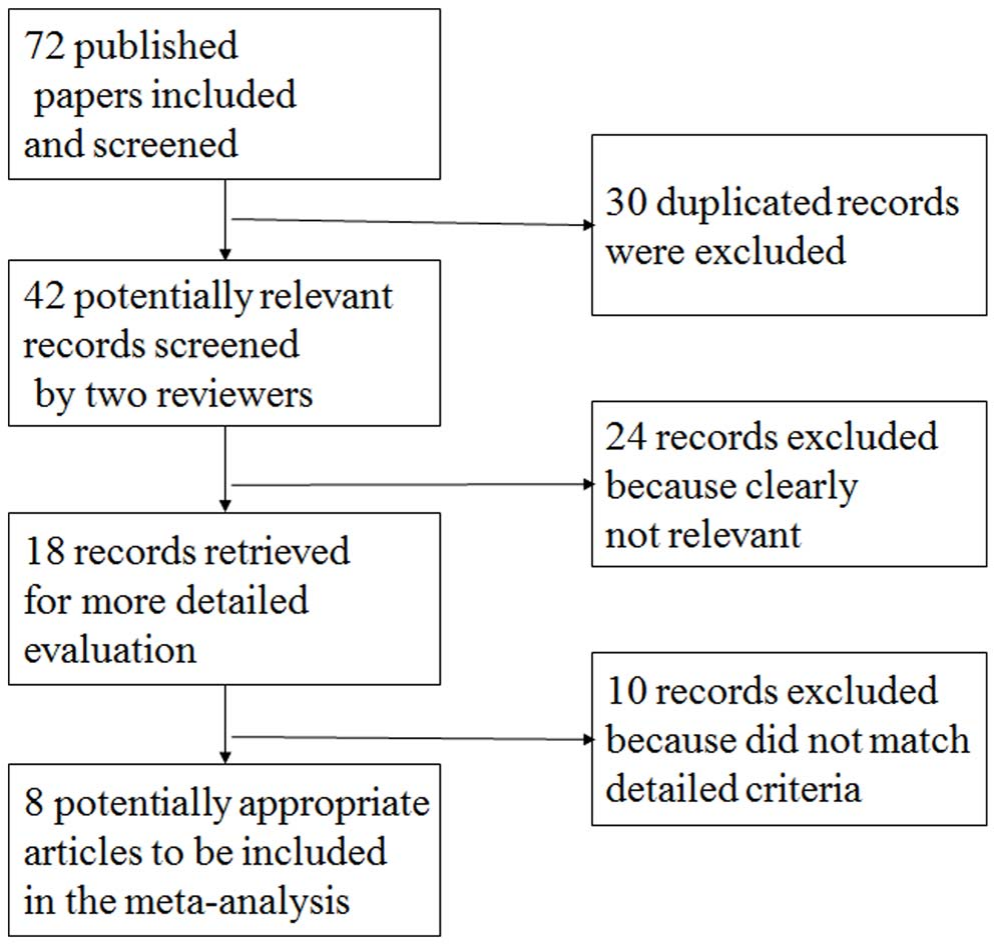
Study flow diagram of search strategy.

Seventy-two papers relevant to the words searched were retrieved (Figure 1). Through the step of screening the title, 30 duplicated articles were excluded with initial assessment. The rest 42 articles were reviewed and an additional 24 trials were excluded because of clearly not relevant, leaving 18 studies for detailed review. Of these, 10 records were excluded because they did not match the detailed criteria. At last, we identified 8 eligible studies, published from 1996 to 2009, that reported on polymorphisms of *APOE* and risk of CRN [6], [24], [25], [26], [34], [35], [36], [37]. Studies were carried out in Japan, Brazil, China, Australia, Finland, USA, UK and Canada. Characteristics of the studies included in the meta-analysis with *APOE* polymorphisms and CRN is provided through Table 1. Table 2 showed the allele frequencies and percentage of *APOE* polymorphism carriers among CRN cases and controls. Appropriate genotyping methods for *APOE* were stated in all studies, all of which was polymerase chain reaction restriction fragment length polymorphism except 1 study [35] using immunoblotting techniques. The deviation from Hardy–Weinberg equilibrium was assessed by the HWE program, and the results indicated that the genotype distribution of control population in most of the eight included studies were in Hardy–Weinberg equilibrium except one study [25].

**Table 1:** Characteristics of the included studies with apolipoprotein E polymorphisms and colorectal neoplasm.

Author/year of publication/country	Enrolment	Case ascertainment	Control definition	Age, y, meanor range	Percentageof adenoma	Percentageof cancer	Colono-scopy
Shinomiya/2001/Japan	1995–1996	Histologically	Polyp-free	Not reported	100	0	Total or partial colonoscopy
Souza/2009/Brasil	2002–2003	Histologically	Neoplasia-free	60.6	0	100	Colonoscopy
Zhongyin/2006/China	2003–2005	Histologically	Healthy individual	68.2	100	0	Colonoscopy
Butler/2001/Australia	Not reported	Not reported	Volunteer	70	100	0	Not reported
Kervinen/1996/Finland	1989–1992	Histologically	Volunteer	64.5	52.53	47.47	Total colonoscopy
Slattery/2005/USA	1997–2001	Not reported	Unaffected control	30–79	0	100	Not reported
Watson/2003/UK	1997–1999	Histologically	Healthy volunteer	55–66	100	0	Sigmoido-scopy
Mrkonjic/2009/Canada	1997–2000	Histologically	Unaffected controls	20–74	0	100	Not reported

**Table 2:** Allele frequencies and percentage of apolipoprotein E polymorphisms carriers among CRN cases and controls.

Study/first author	Ethnicity	Source of controls	Type of lesion	Cases				Controls			
				ε2	ε3	ε4	Total	ε2	ε3	ε4	Total
Shinomiya S, et al.	Asians	Hospital	205 colorectal adenoma	18	296	44	358	25	357	58	440
			69 proximal adenoma	9	113	7	129	25	357	58	440
			110 distal adenoma	9	183	37	229	25	357	58	440
Souza DRS, et al.	Brazilian	Hospital	87 colorectal cancer	11	143	20	174	12	116	18	146
Zhoungyin Z, et al.	Chinese	Hospital	98 colorectal adenoma	17	168	11	196	5	67	8	80
Butler WJ, et al.	Caucasians	Population	219 colorectal cancer	21	266	47	334	31	303	66	400
Kervinen K, et al.	Caucasians	Population	257 colorectal neoplasm	21	426	67	514	13	313	72	398
			122 colorectal carcinoma	7	204	33	244	13	313	72	398
			135 colorectal adenoma	14	202	34	758	13	313	72	398
			81 proximal neoplasm	8	142	12	162	13	313	72	398
			176 distal neoplasm	13	284	55	352	13	313	72	398
Slattery ML, et al.	Mainly Caucasians	Not reported	2333 colorectal cancer	405	3544	697	4646	475	4534	845	5854
			1556 colon cancer	272	2355	475	3102	323	2990	575	3888
			777 rectal cancer	133	1189	222	1544	152	1544	370	1966
Watson MA, et al.	Caucasians	Population	206 colorectal cancer	39	303	70	412	52	550	104	706
			59 proximal adenoma	13	90	15	118	52	550	104	706
			147 distal adenomas	26	213	55	294	52	550	10	706
Mrkonjic M, et al.	Mainly Caucasians	Hospital	906 colorectal cancer	109	2757	206	3072	138	3156	256	3550

### Overall analyses on the association of *APOE* polymorphisms and CRN

**Figure 2:**
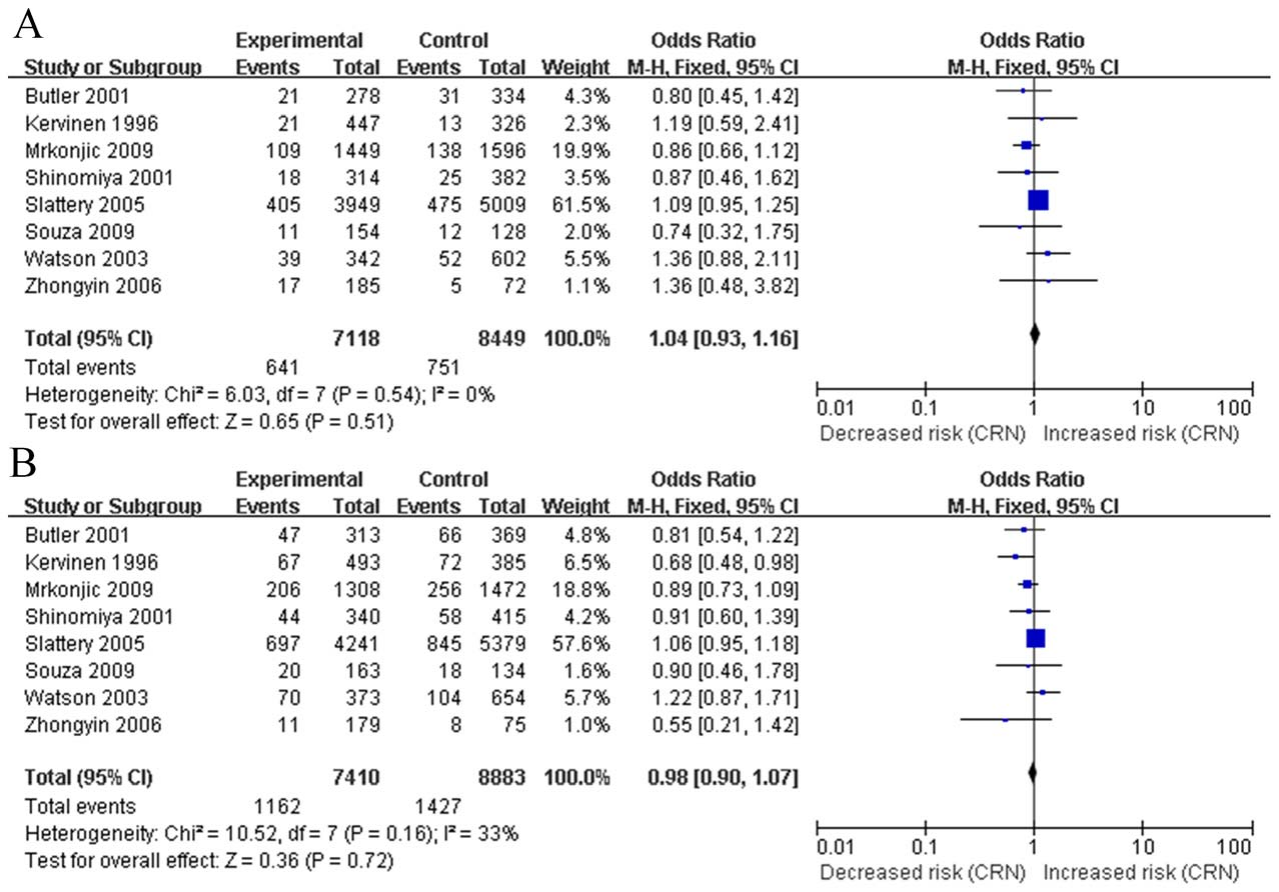
Odds ratio of colorectal neoplasm (CRN) (adenoma and cancer combined) with *APOE* polymorphism for ε2 versus ε3 (A) and ε4 versus ε3 (B).

The meta-analysis of the *APOE* alleles and the risk of CRN was performed firstly. All 8 studies were eligible for assessing the impact of at least one of *APOE* alleles on the CRN risk [6], [24], [25], [26], [34], [35], [36], [37]. Comparison of prevalence of the ε2 vs. ε3 alleles among cases and controls showed no statistically significant heterogeneity between studies (Q = 6.03, p = 0.54, I^2^ = 0%, Figure 2A). The fixed-model was then used. Among the populations in the included studies, the presence of ε2 allele conferred no risk for CRN (OR, 1.04; 95% CI, 0.93 to 1.16; p = 0.51, Figure 2A). In addition, the association of ε4 carriers vs. ε3 alleles between cases and controls was estimated. Because there was no statistical heterogeneity between studies (Q = 10.52, p = 0.16, I^2^ = 33%, Figure 2B), the fixed effects mode was applied. The pooled data indicated presence of the ε4 allele indicated no decreased risk of CRN, in comparison with the ε3 allele (OR 0.98 95% CI 0.90 to 1.07, p = 0.72; Figure 2B). Our data also showed that individuals with the ε2 had similar risk of CRN compared with those with the ε4 (OR 1.04, 95% CI 0.92 to 1.19, p = 0.52). (Figure 2). Results of genotypic models for comparisons of E2, E3 and E4 genotypes in both dominant and recessive models were presented in table 3.

**Table 3:** Comparisons of apolipoprotein E genotype and CRN risk.

Comparisons		Pooled OR (95% CI)	P value	I^2^ (%)
E2 vs E3 for CRN	E2/2 vs E3/3	0.99 (0.56, 1.77)	0.99	0
	E2/3 vs E3/3	1.07 (0.94, 1.22)	0.32	40
	E2/2+E2/3 vs E3/3	1.07 (0.94, 1.22)	0.32	22
	E2/2 vs E2/3+E3/3	1.00 (0.56, 1.77)	0.99	0
E4 vs E3 for CRN	E4/4 vs E3/3	0.93 (0.69, 1.26)	0.64	0
	E4/3 vs E3/3	1.01 (0.91, 1.12)	0.90	41
	E4/4+E4/3 vs E3/3	1.00 (0.90, 1.11)	0.99	40
	E4/4 vs E4/3+E3/3	0.93 (0.69, 1.25)	0.62	0
E2 vs E3 for CRC	E2/2 vs E3/3	0.99 (0.55, 1.78)	0.97	0
	E2/3 vs E3/3	1.07 (0.94, 1.22)	0.32	52
	E2/2+E2/3 vs E3/3	1.07 (0.94, 1.22)	0.33	38
	E2/2 vs E2/3+E3/3	0.99 (0.55, 1.78)	0.98	0
E4 vs E3 for CRC	E4/4 vs E3/3	0.93 (0.69, 1.26)	0.64	0
	E4/3 vs E3/3	1.01 (0.91, 1.13)	0.78	30
	E4/4+E4/3 vs E3/3	1.01 (0.91, 1.13)	0.78	30
	E4/4 vs E4/3+E3/3	0.93 (0.69, 1.25)	0.62	0
E2 vs E3 for CRA	E2/2 vs E3/3	0.87 (0.11, 6.91)	0.89	0
	E2/3 vs E3/3	1.43 (0.69, 2.97)	0.33	0
	E2/2+E2/3 vs E3/3	1.42 (0.70, 2.86)	0.33	0
	E2/2 vs E2/3+E3/3	0.85 (0.11, 6.76)	0.88	0
E4 vs E3 for CRA	E4/4 vs E3/3	0.81 (0.30, 2.18)	0.67	0
	E4/3 vs E3/3	0.70 (0.50, 0.98)	0.04	0
	E4/4+E4/3 vs E3/3	0.71 (0.51, 0.98)	0.04	6
	E4/4 vs E4/3+E3/3	0.88 (0.32, 2.36)	0.79	0
E2 vs E3 for proximal CRN	E2/2 vs E3/3	0.67 (0.03, 16.67)	0.81	0
	E2/3 vs E3/3	1.99 (1.08, 3.68)	0.03	0
	E2/2+E2/3 vs E3/3	0.64 (0.03, 15.81)	0.78	0
	E2/2 vs E2/3+E3/3	1.90 (1.03, 3.49)	0.04	0
E4 vs E3 for distal CRN	E4/4 vs E3/3	0.30 (0.06, 1.58)	0.15	0
	E4/3 vs E3/3	0.70 (0.46, 1.07)	0.10	61
	E4/4+E4/3 vs E3/3	0.64 (0.42, 0.97)	0.04	61
	E4/4 vs E4/3+E3/3	0.32 (0.06, 1.73)	0.19	0
E2 vs E3 for distal CRN	E2/2 vs E3/3	1.08 (0.07, 17.43)	0.96	0
	E2/3 vs E3/3	1.48 (0.89, 2.45)	0.13	0
	E2/2+E2/3 vs E3/3	1.46 (0.89, 2.41)	0.13	0
	E2/2 vs E2/3+E3/3	1.05 (0.06, 16.93)	0.97	0
E4 vs E3 for distal CRN	E4/4 vs E3/3	0.90 (0.66, 1.22)	0.49	82
	E4/3 vs E3/3	1.44 (0.68, 3.08)	0.34	0
	E4/4+E4/3 vs E3/3	0.94 (0.70, 1.27)	0.70	81
	E4/4 vs E4/3+E3/3	12.01 (6.84, 21.10)	0.0001	48

### Overall analyses on the association of *APOE* polymorphisms and CRC

**Figure 3:**
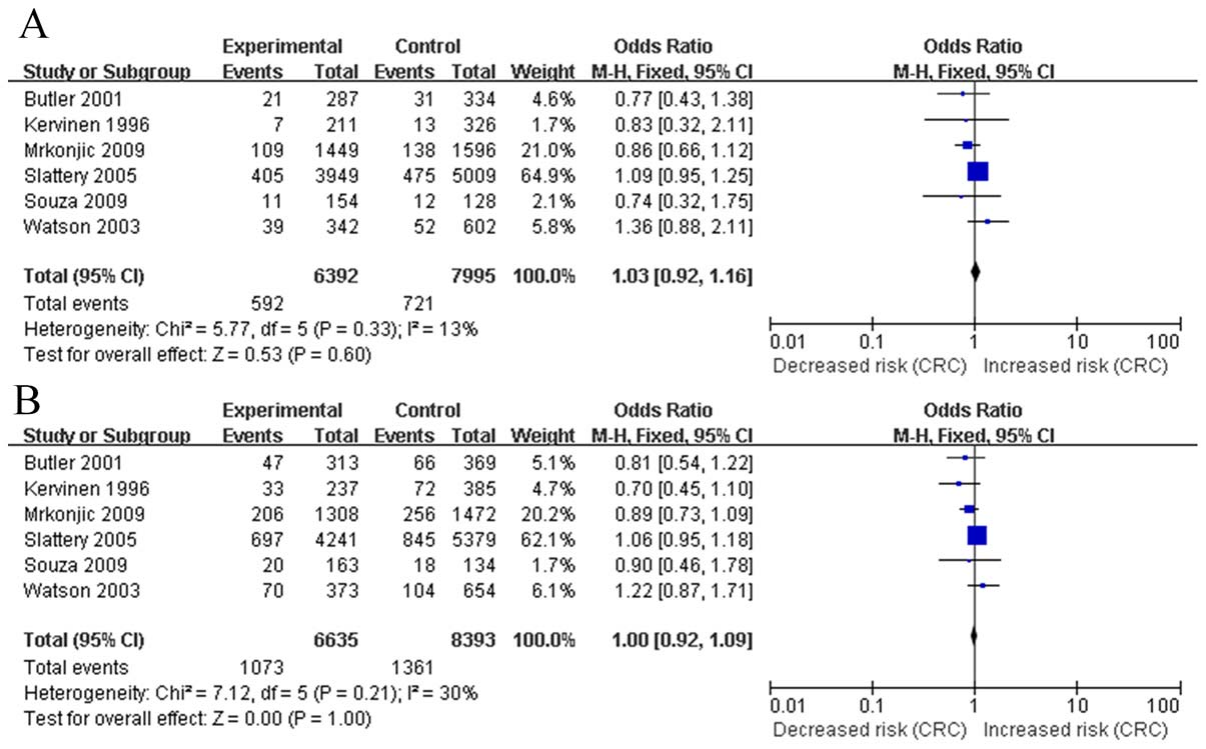
Forest plots of the meta-analysis of associations between alleles of *APOE* polymorphism and CRC (colorectal cancer) risk (A, ε2 versus ε3; B, ε4 versus ε3).

There were a total of 6 studies evaluating the association between *APOE* polymorphisms and CRC. Five studies of the ε2 vs. ε3 alleles were enrolled in this analysis [6], [24], [25], [34], [35], [37]. The combined results based on these 6 studies showed that, compared with those with ε2 alleles, there was no significant risk of CRC of individuals with the ε3 alleles (OR 1.03, 95% CI 0.92 to 1.16, p = 0.60, Figure 3, A). Fixed effects mode was used because there was no heterogeneity between studies (Q = 5.77, p = 0.33, I^2^ = 13%, Figure 3, A). There were 6 studies of the ε4 vs. ε3 alleles were enrolled in this analysis [6], [24], [25], [34], [35], [37]. The pooled data indicated that, compared with those with ε4 alleles, there was no significant risk of CRC of individuals with the ε3 alleles (OR 1.00, 95% CI 0.92 to 1.109, p = 1.00; Figure 3, B). Fixed effects mode was used as there was no heterogeneity between studies (Q = 7.12, p = 0.21, I^2^ = 30%, Figure 3, B). We found that, in comparison with the ε4 carriers, ε2 carriers had a similar risk for CRC development (OR 1.00, 95% CI 0.87 to 1.15, p = 1.00). Table 3 showed the analysis of of E2, E3 and E4 genotypes comparisons in both dominant and recessive models were presented in table 3.

### Overall analyses on the association of *APOE* polymorphisms and CRA

**Figure 4:**
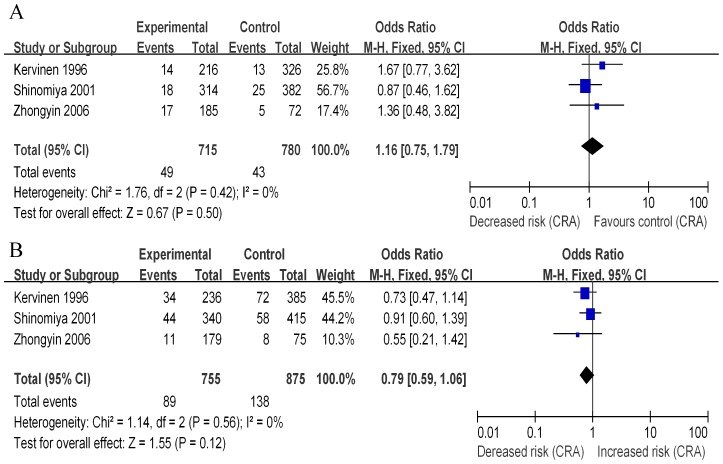
Forest plots of odds ratio with 95% CI for *APOE* polymorphism and CRA (colorectal adenoma) risk (A, ε2 versus ε3; B, ε4 versus ε3).

There were a total of 3 studies of the ε2 vs. ε3 alleles for CRA were enrolled in this analysis [26], [35], [36]. The combined results based on these 3 studies did not provide evidence of significant risk of CRA of individuals with the ε2 alleles when compared with those with ε3 alleles (OR 1.16, 95% CI 0.75 to 1.79, p = 0.50; Figure 4, A). Fixed effects mode was used as there was no heterogeneity between studies (Q = 1.76, p = 0.42, I^2^ = 0%, Figure 4, A). Three studies of the ε4 vs. ε3 alleles for CRA were enrolled in this analysis [26], [35], [36]. The pooled data did not support the concept that individuals with the ε4 alleles presented significant decreased risk of CRA, compared with those with ε3 alleles (OR 0.79, 95% CI 0.59 to 1.06, p = 0.12; Figure 4, B). Fixed effects mode was applied because the absence of heterogeneity between studies (Q = 1.14, p = 0.56, I^2^ = 0%, Figure 4, B). In addition, we found there was no difference in CRA risk among individuals with the ε2 or ε4 genotypes (OR 1.48, 95% CI 0.89 to 2.45, p = 0.13). Results of genotypic models for comparisons of E2, E3 and E4 genotypes in both dominant and recessive models were indicated in table 3.

### Overall analyses on the association of *APOE* polymorphisms and proximal CRN

**Figure 5:**
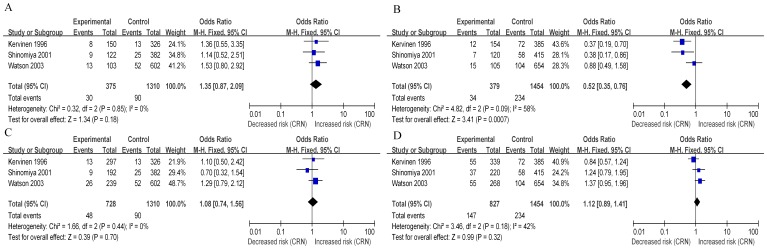
Forest plots of odds ratio with 95% CI for *APOE* polymorphism and proximal CRN (A: ε2 versus ε3, B, ε4 versus ε3) and distal CRN risk (C: ε2 versus ε3, D, ε4 versus ε3).

There were 3 studies with extractable data evaluated the association between *APOE* polymorphisms and proximal CRN [24], [35], [36]. Subgroup analysis based on these 3 studies showed that, compared with those with ε2 alleles, there was no significant risk of proximal CRN of individuals with the ε3 alleles (OR 1.35, 95% CI 0.87 to 2.09, p = 0.18, Figure 5, A). Fixed effects mode was used because there was no heterogeneity between studies (Q = 0.32, p = 0.85, I^2^ = 0%, Figure 5, A). However, data from this subgroup analysis demonstrated the significant decreased risk of proximal CRN of individuals with the ε4 alleles when compared with those with ε3 alleles (OR 0.52, 95% CI 0.35 to 0.76, p = 0.0007; Figure 5, B). Fixed effects mode was used as there was no heterogeneity between studies (Q = 4.82, p = 0.09, I^2^ = 58%, Figure 5, B). In addition, data of genotypic models for comparisons of E2, E3 and E4 genotypes in both dominant and recessive models were presented in table 3.

### Overall analyses on the association of *APOE* polymorphisms and distal CRN

Subgroup analysis was also performed to compare prevalence of the ε2 vs. ε3 alleles among distal CRN cases and controls. Pooled data from the available 3 studies [24], [35], [36] showed the presence of ε2 allele conferred no risk for distal CRN (OR, 1.08; 95% CI, 0.74 to 1.56; p = 0.70, Figure 5, C). The fixed-model was then used as there was no statistically significant heterogeneity between studies (Q = 1.66, p = 0.44, I^2^ = 0%, Figure 5, C). In addition, the association of ε4 carriers vs. ε3 alleles between distal CRN cases and controls was estimated. Because there was no statistical heterogeneity between studies (Q = 3.46, p = 0.18, I^2^ = 42%, Figure 5, D), the fixed effects mode was applied. The pooled data indicated presence of the ε4 allele indicated no decreased risk of distal CRN, in comparison with the ε3 allele (OR 1.12, 95% CI 0.89 to 1.41, p = 0.32; Figure 5, D). Additionally, genotypic analysis for comparisons of E2, E3 and E4 genotypes in both dominant and recessive models were presented in table 3.

### Publication bias

**Figure 6:**
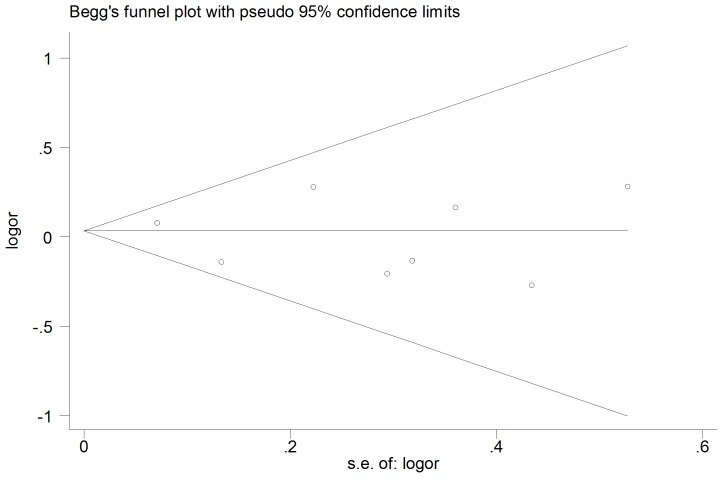
Funnel plot of the meta-analysis.

We also evaluated the publication bias by testing funnel plots for obvious asymmetry. No publication bias was found from either visualization of the funnel plot or statistics of. Our data indicated there was no statistical evidence of publication bias (Egger’s, P = 0.7, Begg’s P = 0.805) (Figure 6).

## Discussion

Eight eligible studies at last included in this meta-analysis, and 5 studies of them suggested *APOE* ε4 is a protective factor. In this meta-analysis, we used a total of 9243 subjects and 4310 CRN cases which including 438 patients with CRA, and 3873 patients with CRC from 8 publications to evaluate the association of *APOE* gene polymorphism with CRN. This meta-analysis suggested that having an *APOE* allele doesn’t increase the risk of CRN. Although *APOE* ε4 has been considered to be a protective factor in CRN [25], [34], our results indicated there was no association between a ε4 allele and CRA development.

*APOE* seems to be involved in immunoregulation [38] and inhibiting endothelial cell proliferation [20], which may directly affect the adenoma to carcinoma process. It was suggested that *APOE* may influence CRC development through three potential path ways: cholesterol and bile metabolism, triglyceride and insulin regulation, and the prolonged inflammation [37]. Due to different affinity to its receptors, *APOE* can influence hepatic cholesterol processing by enhancing cholesteryl ester hydrolysis [39], and people with the allele ε4 were found to have an increased intestinal absorption of cholesterol [40] and to have a lower biliary excretion of deoxycholic acid [41]. It was speculated *APOE* ε4, which is associated with more intracellular release of free than that of ε3 [42] and lower concentrations of fecal bile acids in the gastrointestinal tract, has its protective role against CRC [35].

The *APOE* ε4 allele appears to be associated with an increased risk of gallstones and breast cancer [43], [44]. Our present data indicated that the *APOE* gene polymorphisms were similar between patients with CRN and controls. Our data also demonstrated *APOE* ε4 did not affect the overall risk for CRA, there was not a protective effect in patients with ε4 when compared to those with ε3. Despite the genetic factors has been suggested to be important for the susceptibility to CRN, other factor like racial differences may also play a role. First, genetic heterogeneity may be a reason for the conflicting results. In people of European ancestry, *APOE* genotype showed a positive dose-response association with LDL-C [45] while study of Brazilian individuals indicated that the presence of the ε4 genotype may be a protective effect against CRC [25]. In addition, the allele ε4 is much less frequent in Japanese than in Caucasians, and it was reported Finns seem to have a particularly high frequency of the allele ε4 [46], [47]. In our meta-analysis, the included 8 studies are from Japan, Brazil, China, Australia, Finland, USA, UK, and Canada respectively on evaluating the *APOE* polymorphisms in relation to CRN. In addition, our data indicated the contribution of *APOE* polymorphisms to CRN susceptibility varies in different studies. For ethnic diversity, distinct environmental factors and eating habits characterize populations, analyze the allelic and genotypic distributions of the *APOE* and their association with CRA or CAC should characterize the histories and habits of people.

We also evaluated association of genetic variants of *APOE* with proximal and distal CRN. Three of the 8 included studies involving evaluated the presence of *APOE* polymorphisms to different parts of the colorectal tumors [24], [35], [36]. Although *APOE* ε4 did not affect the overall risk for CRN, there was a trend towards a protective effect in patients with right-sided cancer when compared to those with left-sided carcinoma [34], [48]. However, the degree of this protection was less prominent reported by Kervinen et al. from Finland [35]. Our meta-analysis data demonstrated, compared with those carry *APOE* ε3 alleles, persons with *APOE* ε4 genotype have significant decreased risk suffering from proximal CRN but not from distal CRN. Several reasons for the protective association between the allele ε4 of *APOE* and proximal colon adenomas have been reported in past years. A proposed mechanism involving in this different effect between proximal and distal CRN is the decreased levels of fecal bile acids which may result in relative lower levels of cell proliferation in the proximal colon [41]. A potential mechanism for this effect is the low levels of fecal bile acids which may resulting in lower levels of epithelial proliferation in the proximal colon [41]. Serum cholesterol acids are positively related to the risk of CRN [49], [50] and patients with colorectal adenomas indicated high serum deoxycholic acid levels [22]. However, in patients with the ε4 allele of *APOE*, the levels of biliary deoxycholic acid are relatively low [41], which may be associated with the low incidence of adenoma and carcinoma. This has been confirmed by the results that *APOE* has the ability in inhibiting endothelial proliferation [20] and *APOE* shows its ability in immunoregulation [51]. It seems that the alterations in luminal cholesterol delivery and fecal bile acid are involved in the protective association of the allele ε4 and proximal CRN development [35], [52]. *APOE* genotypes has been reported implicated in the breast cancer [53]. *APOE* ε4 allele is found to be a low-penetrant risk factor for development of breast cancer [43]. The possible biological mechanisms of the association between *APOE* ε4 genotype and carcinoma of the proximal colon and breast is subjects carrying *APOE* ε4 genotype less than half of the risk of tumor cell proliferation [20].

CRN incidences differ considerably between Western and non-Western countries. In recent years, a dramatic increase in CRC incidence has been reported in several Asian countries. Two studies from Asia included in our meta-analysis [26], [36] found *APOE* ε4 was protective factor for CRN. Immigration studies have suggested that environmental factors rather than genetic susceptibility are primarily responsible for the secular trends of CRC incidence rates and international variability. It is more likely that the interaction of genetic susceptibility and environmental factors is the causation of colorectal carcinomas and adenomas. Therefore, not only the main effect of a gene but also the influence of gene-environment interactions on cancer risk are important from the public health perspective [54].

We must confess that some limitations of this study were still inherited from the published studies. First, many of the studies included in the analysis were published a decade ago, recent advance of this issue is limited. Second, selection bias may play a role in this meta-analysis. Third, due to the limited patients included in this study, it was impossible for us to perform the subgroup analysis. Last but not the least is that account of potential confounding factors which might be associated with the risk of CRN.

In conclusion, the pooled data showed no significant association between *APOE* genotype and CRN. However, compared with those carry *APOE* ε3 alleles, persons with *APOE* ε4 genotype have significant decreased risk suffering from proximal CRN but not from distal CRN. Due to the small number of studies addressing the association of *APOE* polymorphisms and CRN, the conclusion whether *APOE* ε4 and ε2 increase or decrease the susceptibility to CRN requires further investigation. The mechanism of the involvement of *APOE* in carcinogenesis is still not clear and further studies with larger samples are necessary to confirm this in population.

## Supporting Information

Checklist S1 PRISMA Checklist.
(DOC)
